# Age-period-cohort analysis of infectious disease mortality in urban-rural China, 1990–2010

**DOI:** 10.1186/s12939-016-0343-7

**Published:** 2016-03-31

**Authors:** Zhi Li, Peigang Wang, Ge Gao, Chunling Xu, Xinguang Chen

**Affiliations:** School of Social and Behavioral Sciences, Nanjing University, Nanjing, China; Global Health Institute, Wuhan University, Wuhan, China; School of Basic Medical Sciences, Peking University, Beijing, China; Department of Epidemiology, University of Florida, FLorida, USA

**Keywords:** Infectious disease, Mortality, APC Model, IE, Urban- rural China

## Abstract

**Background:**

Although a number of studies on infectious disease trends in China exist, these studies have not distinguished the age, period, and cohort effects simultaneously. Here, we analyze infectious disease mortality trends among urban and rural residents in China and distinguish the age, period, and cohort effects simultaneously.

**Methods:**

Infectious disease mortality rates (1990–2010) of urban and rural residents (5–84 years old) were obtained from the China Health Statistical Yearbook and analyzed with an age-period-cohort (APC) model based on Intrinsic Estimator (IE).

**Results:**

Infectious disease mortality is relatively high at age group 5–9, reaches a minimum in adolescence (age group 10–19), then rises with age, with the growth rate gradually slowing down from approximately age 75. From 1990 to 2010, except for a slight rise among urban residents from 2000 to 2005, the mortality of Chinese residents experienced a substantial decline, though at a slower pace from 2005 to 2010. In contrast to the urban residents, rural residents experienced a rapid decline in mortality during 2000 to 2005. The mortality gap between urban and rural residents substantially narrowed during this period. Overall, later birth cohorts experienced lower infectious disease mortality risk. From the 1906–1910 to the 1941–1945 birth cohorts, the decrease of mortality among urban residents was significantly faster than that of subsequent birth cohorts and rural counterparts.

**Conclusions:**

With the rapid aging of the Chinese population, the prevention and control of infectious disease in elderly people will present greater challenges. From 1990 to 2010, the infectious disease mortality of Chinese residents and the urban-rural disparity have experienced substantial declines. However, the re-emergence of previously prevalent diseases and the emergence of new infectious diseases created new challenges. It is necessary to further strengthen screening, immunization, and treatment for the elderly and for older cohorts at high risk.

## Background

Since the founding of the People’s Republic of China, with the improvement of living standards and the development of medical and health services, the health of Chinese residents has been greatly improved. Disease patterns have also undergone tremendous change in that chronic rather than infectious diseases have become the leading cause of death [1–3]. Most infectious diseases have been eliminated or controlled at a low endemic level since the 1990s. However, upon entering the 21st century, China is facing a complex scenario involving the re-emergence of previously prevalent diseases and the emergence of new infectious diseases [4, 5]. Entities that continue to pose serious threats to lives and health include HIV/AIDS, sexually transmitted diseases (e.g., syphilis, gonorrhea), viral hepatitis, tuberculosis, and zoonoses (e.g., rabies, SARS, HPAI, and H1N1). Recent infectious disease outbreaks such as SARS, HPAI, and H1N1 remind us that any slackness in prevention and control will probably trigger disease re-emergence [5]. Infectious diseases not only harm lives and health, but also lead to economic losses and social instability. Considering the increasingly close contacts between China and the rest of the world, infectious diseases are no longer just local issues: if not quickly detected and contained, they may transform into national and even global pandemics [4]. Therefore, the scientific measurement of trends in China’s infectious diseases is critical.

When researching the development trend of infectious diseases in China, special attention should be paid to the unbalanced development between urban and rural areas. In addition to basic living standards, there is also a considerable gap in health services between them. Therefore, the capacity for the prevention and control of infectious diseases in these settings is different. In China, most medical resources are concentrated in urban areas, including the best medical institutions, facilities, and personnel [6]. In 2012, the health expenditure per capita was 2,969 yuan for urban residents and only 1,056 yuan for rural residents; the former is 2.8 times that of the latter [7]. Urban and rural residents are also subject to different health insurance schemes and a widening income gap. Thus, with the rapid increase of health care costs, their medical burdens are different [1, 8–11]. The fourth National Health Services Survey in 2008 showed that the proportions of urban and rural residents who were ill but did not take any therapeutic measures within the last two weeks were 6.4 % and 12.4 % respectively and the proportions of residents untreated for economic reasons were 23.2 % and 30.6 %. The values of both indicators were significantly lower among urban residents than rural residents [12].

A number of studies on infectious disease trends in China already exist. These studies investigated either a variety of infectious diseases [4, 5] or focused on a specific entity. Studied disease entities include HIV/AIDS [13–17], sexually transmitted diseases [18–20], viral hepatitis [21–23], tuberculosis [24–26], rabies [27, 28], and others [29–31]. They focused on comparing infectious disease trends across years, and more detailed studies cared age-specific trends. One disadvantage of these studies is that they did not distinguish the age, period, and cohort effects^1^ simultaneously. These should be distinguished not only because of their differences, but also because failing to do so leads to bias and makes reliable conclusions difficult to get [32, 33], thereby affecting trend attribution. Although some studies have started using APC models to analyze infectious disease trends outside mainland China [34–37], the model estimation method used in these studies has some disadvantages (see methods section). To overcome shortcomings of former studies, we used the IE [38] to estimate the model. Compared with other methods, this estimation method can not only solve the problem of model identification, but also provide unbiased and relatively efficient estimation results [39, 40].

Here, we aim to analyze infectious disease mortality trends (1990–2010) of urban and rural Chinese residents (5–84 years old) using the APC model and to distinguish the age, period, and cohort effects simultaneously. First, through a descriptive analysis of age-specific mortality rates, we will obtain a visual impression of age, period, and birth cohort effects as well as demonstrate the need to distinguish these three effects. We then use the IE method to estimate the coefficients of the age, period, and cohort variation in infectious disease mortality.

## Methods

The age-specific infectious disease mortality rates used in this study were obtained from the China Health Statistical Yearbook. Mortality rates were collected for 5-year age groups. The APC models requires a uniform age group interval [41], and therefore the 0, 1–4, and above 85 age groups were not included in the analysis. Since the mortality rates were organized in 5-year age groups, data with a 5-year interval were used according to the requirement of the APC model at the collective level. In view of data availability and the APC model requirements, this paper analyzes the infectious disease mortality of Chinese urban and rural residents from 1990 to 2010. As data of year 2000 were not available, we used the mean values of the 1999 and 2001 mortality to represent them.

Age-specific infectious disease mortality rates are summarized in the *a × p* table with *a* age groups and *p* time periods. The diagonal elements of the matrix denote *c (c = a + p-1)* cohorts. The oldest birth cohort observations appear in the oldest age group and earliest years, and the youngest appear in the youngest age group and latest years. The data used in this study comprise 16 5-year age groups ranging from 5–9 to 80–84 and 5 period points with a 5-year interval from 1990 to 2010. These yielded 20 successive 5-year birth cohorts of which the oldest cohort was born in 1906–1910 (80–84 years old in 1990) and the youngest cohort was born in 2001–2005 (5–9 years old in 2010).

### Methods

When analyzing infectious disease mortality, we constructed an APC model using the Poisson log-linear model [42]:

1 $$ \log \left({r}_{ijk}\right)= \log \left(\frac{d_{ijk}}{n_{ijk}}\right)=\mu +{\alpha}_i+{\beta}_j+{\gamma}_k $$

In this model, r_ijk_ denotes the expected death rate of the i-th age group in the j-th year (k-th birth cohort); d_ijk_ denotes the expected number of deaths and is assumed to be distributed as a Poisson variate; n_ijk_denotes the number of people exposed at risk; μ denotes the intercept or adjusted mean; α_i_ denotes the effect of the i-th age group (i = 1,……,a); β_j_ denotes the effect of the j-th year (j = 1,……,p); and γ_k_ denotes the effect of k-th birth cohort (k = 1,……,c), where c = a + p-1.

For a long time, the use of Eq. (1) to construct the APC model was hindered by the so-called “model identification problem”: Because there is a linear relationship between age, period, and birth cohort (Birth Cohort = Period - Age), a definite solution of the model cannot be obtained using traditional estimation methods such as the ordinary least squares method. Although researchers have made various attempts to resolve the problem, such as using a reduced model containing two of the factors or constraining the model parameters [43], no satisfactory solution was identified totally. The use of a reduced model ignores the third effect, while constraining the parameters depends heavily on certain assumptions that are difficult to verify [39, 40]. Here, we use IE [38] to construct the model, which not only solves the problem of model identification but also provides unbiased and relatively efficient estimation results [39, 40].

## Results and discussion

### Descriptive analysis

**Fig. 1:**
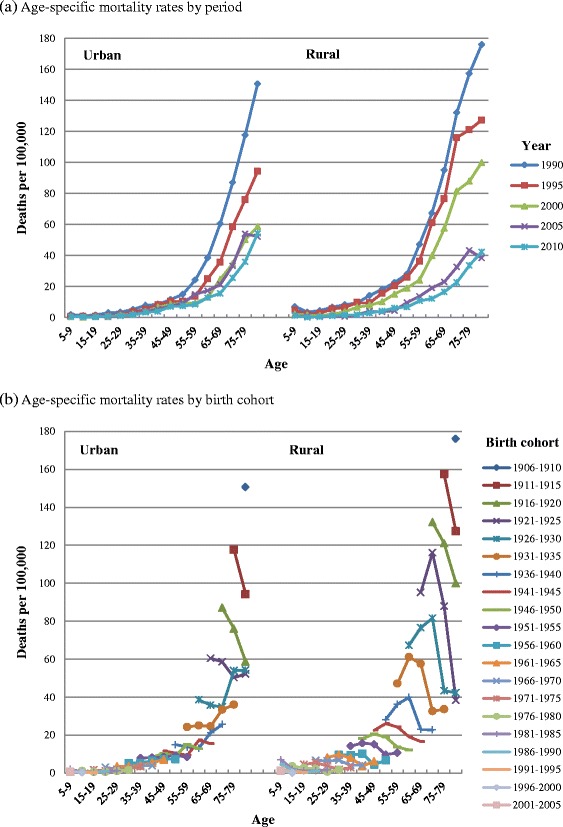
Age-specific infectious disease mortality rates of Chinese urban and rural residents, 1990–2010. **a** Age-specific mortality rates by period. **b** Age-specific mortality rates by birth cohort

**Fig. 2:**
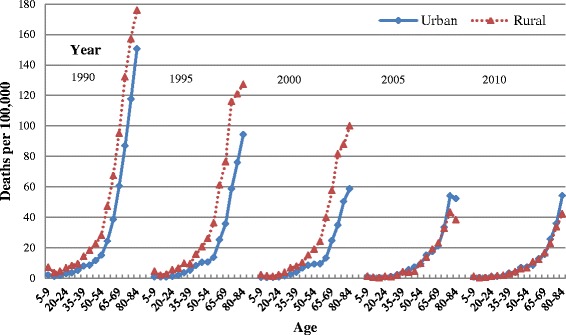
Age-specific infectious disease mortality rates of Chinese urban and rural residents (urban-rural disparity comparison), 1990–2010

To obtain a visual impression of the age, period, and birth cohort effects of infectious disease mortality and demonstrate the need to distinguish these three effects, we analyzed age-specific mortality rates by period (Fig. 1a) and cohort (Fig. 1b). Fig. 1a shows that mortality is relatively high at age 5–9 but reaches a minimum in adolescence (age 10–19) before rising with age. However, the age pattern in Fig. 1b is quite different from that in Fig. 1a, showing that mortality might not increase with age but instead decline for some birth cohorts. From 1990 to 2010, the infectious disease mortality of urban and rural residents continuously declined, except that the mortality of urban residents showed no apparent decline between 2000 and 2005. By contrast, the mortality of rural residents declined rapidly during 2000 to 2005. During 1990 to 2000, the mortality of rural residents was higher than that of urban residents; it was during 2000 to 2005 that the mortality gap between urban and rural residents shrank rapidly, with mortality rates tending to be convergent with each other (Fig. 2). With few exceptions, later birth cohorts showed lower mortality.

Although comparisons of age-specific mortality are helpful for achieving some insight into age, period, and cohort effects, the obvious disadvantage of this approach is that it cannot distinguish these three effects simultaneously. In other words, the effects will be confounded, making reliable conclusions difficult to obtain [44]. In Fig. 1a, for a given year, the older age group is from an earlier birth cohort, thereby confounding the age and birth cohort effects. For a given age group, those alive in later years also belong to later birth cohorts, thereby confounding the period and cohort effects. Similarly, in Fig. 1b, for a given birth cohort, the older age group was also alive in later years, thereby confounding the age and period effects. For a given age group, the later birth cohorts were also alive in later years, thereby confounding the birth cohort and period effects. In Fig. 1a, b, it is because the age effects are confounded with the birth cohort and period effects respectively that we observe inconsistent age effects. In addition, although comparisons of age-specific mortality can provide a qualitative impression of age, period, and birth cohort effects, it is unable to provide a more accurate quantitative assessment [45].

### Age, period, and birth cohort effects

**Fig. 3:**
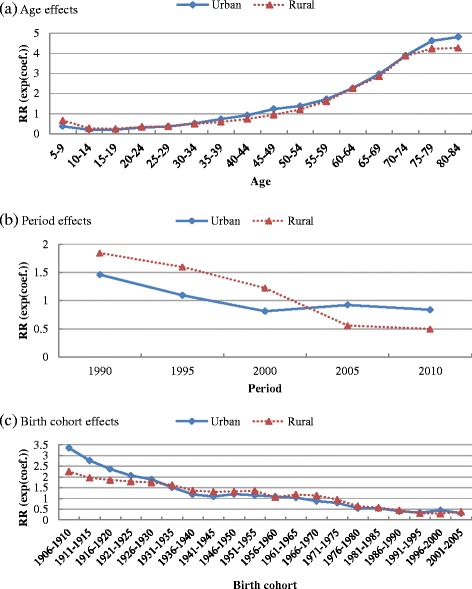
Age, period and birth cohort effects of infectious disease mortality of Chinese urban and rural residents, 1990–2010. **a** Age effects. **b** Period effects. **c** Birth cohort effects

To distinguish the age, period, and cohort effects simultaneously, this study used the APC model (IE) to analyze infectious disease mortality trends (1990–2010) of Chinese urban and rural residents (5–84 years old). Table 1 shows the estimated coefficient, standard error, and other information. To compare the relative mortality risk across ages, periods, and birth cohorts, we calculated the exponential value of the estimated coefficients (exp(coef.) = e^coef.^) (Table 1). Specifically, this value denotes the mortality relative risk (RR) of a particular age, period, or birth cohort relative to average levels [46, 47]. To grasp age, period, and cohort effect trends more intuitively, we plotted Fig. 3 based on the exponential value.

**Table 1:** APC analysis of infectious disease mortality of Chinese urban and rural residents, 1990–2010

	Urban			Rural		
	Coef.	S.E.	Exp (Coef.)	Coef.	S.E.	Exp (Coef.)
Intercept	−9.659^***^	0.047		−9.218^***^	0.043	
Age						
5–9	−0.999^***^	0.201	0.368	−0.415^**^	0.141	0.660
10–14	−1.615^***^	0.223	0.199	−1.296^***^	0.160	0.274
15–19	−1.584^***^	0.204	0.205	−1.425^***^	0.150	0.241
20–24	−1.178^***^	0.162	0.308	−1.055^***^	0.122	0.348
25–29	−1.023^***^	0.145	0.360	−1.004^***^	0.115	0.366
30–34	−0.663^***^	0.126	0.515	−0.701^***^	0.104	0.496
35–39	−0.309^**^	0.107	0.734	−0.543^***^	0.094	0.581
40–44	−0.082	0.094	0.921	−0.300^***^	0.082	0.741
45–49	0.210^**^	0.080	1.234	−0.056	0.071	0.946
50–54	0.322^***^	0.070	1.380	0.188^**^	0.061	1.207
55–59	0.545^***^	0.058	1.725	0.487^***^	0.050	1.627
60–64	0.822^***^	0.049	2.275	0.820^***^	0.043	2.270
65–69	1.090^***^	0.047	2.974	1.051^***^	0.043	2.861
70–74	1.359^***^	0.052	3.892	1.356^***^	0.049	3.881
75–79	1.532^***^	0.062	4.627	1.443^***^	0.059	4.233
80–84	1.573^***^	0.076	4.821	1.452^***^	0.073	4.272
Year						
1990	0.378^***^	0.037	1.459	0.612^***^	0.033	1.844
1995	0.089^***^	0.026	1.093	0.469^***^	0.023	1.598
2000	−0.208^***^	0.025	0.812	0.200^***^	0.021	1.221
2005	−0.080^**^	0.029	0.923	−0.582^***^	0.031	0.559
2010	−0.178^***^	0.040	0.837	−0.698^***^	0.040	0.498
Birth cohort						
1906–1910	1.210^***^	0.091	3.353	0.813^***^	0.081	2.255
1911–1915	1.016^***^	0.073	2.762	0.674^***^	0.063	1.962
1916–1920	0.863^***^	0.059	2.370	0.621^***^	0.050	1.861
1921–1925	0.726^***^	0.048	2.067	0.581^***^	0.041	1.788
1926–1930	0.636^***^	0.042	1.889	0.557^***^	0.036	1.745
1931–1935	0.413^***^	0.046	1.511	0.486^***^	0.041	1.626
1936–1940	0.170^**^	0.058	1.185	0.308^***^	0.052	1.361
1941–1945	0.083	0.071	1.087	0.271^***^	0.064	1.311
1946–1950	0.183^*^	0.083	1.201	0.279^***^	0.077	1.322
1951–1955	0.140	0.098	1.150	0.304^***^	0.089	1.355
1956–1960	0.080	0.113	1.083	0.055	0.105	1.057
1961–1965	0.032	0.127	1.033	0.167	0.114	1.182
1966–1970	–0.132	0.143	0.876	0.124	0.122	1.132
1971–1975	−0.236	0.159	0.790	−0.060	0.135	0.942
1976–1980	−0.606^**^	0.189	0.546	−0.451^**^	0.151	0.637
1981–1985	−0.623^***^	0.181	0.536	−0.550^***^	0.137	0.577
1986–1990	−0.890^***^	0.240	0.411	−0.795^***^	0.171	0.452
1991–1995	−1.100^***^	0.320	0.333	−1.183^***^	0.240	0.306
1996–2000	−0.795^*^	0.318	0.452	−1.219^***^	0.334	0.296
2001–2005	−1.170^*^	0.526	0.310	−0.982^*^	0.409	0.375
Deviance (df = 42)	6.931			8.755		
AIC	4.794			5.277		
BIC	−177.114			−175.291		

*Note*: ^*^ *p* < 0.05, ^**^ *p* < 0.01, ^***^ *p* < 0.001

#### Age effects

The infectious disease mortality of Chinese residents is relatively high at age 5–9, reaches a minimum in adolescence (age group 10–19), then continues to rise with age, with the growth rate gradually slowing down from approximately age 75 (Fig. 3a). For example, among rural residents, the lowest mortality occurs at age group 15–19 and the risk decreases by 64 % (1–0.241/0.660 ≈ 0.635) compared with age group 5–9. Mortality RR then persistently increases by about 17 times (4.272/0.241–1 ≈ 16.726) until age group 80–84, with the growth rate of mortality declining from age group 70–74. Compared with urban residents, rural residents’ mortality rates at age group 5–9 were much higher than the minimum rate in adolescence, while the growth rate of mortality after age group 70–74 declined more significantly. As seen in Figs. 1a and 2, the mortality of children aged 5–9 in rural areas was higher than that of urban children before the year 2000, but this gap gradually disappeared after 2000. Before the year 2000, the mortality of rural residents aged 70–84 was higher than that of urban residents while an opposite trend started after 2000.

#### Period effects

From 1990 to 2000, the infectious disease mortality RR of Chinese residents declined rapidly, by 44 % (1–0.812/1.459 ≈ 0.443) in urban residents and 34 % (1–1.221/1.844 ≈ 0.338) in rural residents (Fig. 3b). From 2000 to 2005, there was a great difference in mortality trends between urban and rural residents, with urban mortality RR increasing by 14 % (0.923/0.812–1 ≈ 0.137) and rural mortality RR decreasing by 54 % (1–0.559/1.221 ≈ 0.542). As mentioned previously, it was during this period that the mortality gap between urban and rural residents substantially narrowed. From 2005 to 2010, the mortality of urban and rural residents declined though at a slower pace. By 2010, urban residents’ mortality was nearly equivalent to the value of year 2000, while the mortality RR of rural residents decreased by 59 % (1–0.498/1.221 ≈ 0.592) during this period.

#### Birth cohort effects

Among Chinese residents, later birth cohorts experienced lower infectious disease mortality, with the exception of some individual cohorts (Fig. 3c). From the 1906–1910 to the 1941–1945 birth cohorts, the decrease of mortality among urban residents was significantly faster than that of subsequent birth cohorts and rural counterparts (the mortality RR among urban residents declined by 68 % [1–1.087/3.353 ≈ 0.676] while that among rural counterparts declined by 42 % [1–1.311/2.255 ≈ 0.419]). It can be seen from Fig. 1a that the rapid decline in the mortality of urban cohorts is partly due to the combination of two factors: (1) the decline of urban mortality was mainly concentrated from 1990 to 2000; (2) the decline is more significant in middle-aged and elderly people and becomes more obvious with age. Thus, birth cohorts who were right at the middle-aged and elderly stage (namely, from 1906–1910 to 1941–1945 birth cohorts) experienced a more significant decline. For the 1946–1950 to 2001–2005 birth cohorts, the decline rates of urban mortality approached those of rural counterparts. For birth cohorts from 1906–1910 to 2001–2005, mortality RR of urban and rural residents decreased by 91 % (1–0.310/3.353 ≈ 0.908) and 83 % (1–0.375/2.255 ≈ 0.834), respectively.

## Conclusions

In the current study, we analyzed infectious disease mortality trends (1990–2010) of urban and rural Chinese residents (5–84 years old) using the APC model and to estimate the age, period, and cohort effects simultaneously. The infectious disease mortality of Chinese residents is relatively high at age group 5–9, reaches a minimum in adolescence (age group 10–19), and then continues to rise with age, with the growth rate gradually slowing down from approximately age 75. The Chinese population is aging rapidly: from 2013 to 2050, the proportion of people aged over 60 will increase rapidly from 13.9 to 32.8 % while the proportion of people aged over 80 will increase from 1.6 to 6.5 % [48]. With the rapid aging of the Chinese population, the prevention and control of infectious disease in elderly people will present a greater challenge. Due to decreased immunity, malnutrition, physiological changes, and many other reasons, the elderly are not only more vulnerable to infectious diseases, but the diseases tend to be more severe and more difficult to treat [49–52]. Therefore, in addition to the prevention and control of infectious diseases in women and children, it is necessary to further strengthen screening, immunization, and treatment for the elderly at high risk.

From 1990 to 2010, the infectious disease mortality of Chinese residents has indeed experienced a substantial decline, except for a slight increase among urban residents from 2000 to 2005. This is largely due to the continuous improvement of living standards and the rapid development of health services. Factors including economic development, scientific and technological progress, as well as the government’s strong commitment and the establishment of an infectious disease surveillance and response system played an important role [4]. In contrast to the slight rise among urban residents during 2000 to 2005, the mortality of rural residents declined rapidly. It was during this period that the mortality gap between urban and rural residents substantially narrowed. This shows that the increased investment in rural health services [6] following the SARS outbreak in 2003 was indeed effective. It is especially worth noting that there was a nationwide expansion of Directly Observed Treatment, Short-Course from 2000 to 2005, which was quickly promoted after SARS [53]. During that period, the tuberculosis mortality rate in rural areas declined rapidly from 7.31/100,000 in 2000 to 2.89/100,000 in 2005 [7]. However, the slight increase in mortality among urban residents during 2000 to 2005 as well as the slowing pace of the decline in mortality among urban and rural residents from 2005 to 2010 indicate that the re-emergence of previously prevalent diseases and the emergence of new infectious diseases created new challenges for China’s prevention and control of infectious disease [4, 5]. Of course, this may be partly associated with the perfection of the infectious disease surveillance system in China [4, 54].

Among Chinese residents, later birth cohorts experienced lower infectious disease mortality. This may be largely due to the better living and health conditions of younger cohorts and their exposure at younger ages under these favorable circumstances [32, 34]. Meanwhile, for birth cohorts from 1906–1910 to 1941–1945, the decrease of mortality among urban residents was significantly faster than that of subsequent birth cohorts and rural counterparts. This is partly because the rapid decline in urban mortality more obviously benefited birth cohorts who were right at the middle-aged and elderly stage. Of course, it may also relate to advanced medical development and antibiotics usage during these birth cohorts’ early life [32]. Further studies are needed to fully understand this difference. Given that infectious disease mortality in older cohorts is still very high, in addition to the prevention and control of infectious disease in younger cohorts, it is necessary to further strengthen screening, immunization, and treatment for older cohorts at high risk. The rapid decline of mortality from 1906–1910 to 1941–1945 birth cohorts supports this viewpoint, and some recent studies also indicate that a prevention and control approach based on birth cohort can be cost-effective [55–57]. In the United States, the prevalence of hepatitis C is highest in the 1945–1965 birth cohorts, but many in these cohorts do not realize they are infected; these studies suggest they should be screened and treated.

Although we analyzed the age, period, and cohort effects of Chinese urban and rural residents’ infectious disease mortality, we could not further evaluate mortality trend differences by disease-specific or gender-specific due to data availability. This analysis is important because it can help clarify the factors that influence infectious disease trends and to locate people at high risk with greater accuracy [32]. For example, in contrast to tuberculosis and hepatitis B, AIDS mortality is higher at middle age, has grown steadily in recent years, and may be trending upward among younger birth cohorts [35]. Also, this analysis can help compare the results with those of existing studies on infectious diseases in China as well as studies in other countries. Meanwhile, further research should distinguish the internal difference in disease mortality trends of urban and rural residents. Since the reform and opening-up policy^2^, the disparity within urban and rural areas continues to expand, and regional disparity is particularly worrisome [58]. This disparity makes a big difference in the ability of various regions to cope with disease, and is evident in the proportions of people who were ill but did not take any therapeutic measures within the last two weeks. In 2008, the proportion in big cities was 19.1 % while that in small cities was as high as 27.3 %; the proportion in first class rural areas was 28.5 % and that in fourth class rural areas was 35.5 % [12]. For residents who are from Central and Western regions, especially the rural areas of these regions, poverty is still an important factor threatening their health; conversely, diseases are also an important reason for their return to poverty [8].

## Footnotes

1 Age effects refer to the risk differences between different age groups. As people age, changes in physiological status, social roles and life experiences will bring these differences. Period effects refer to the risk differences between different years. These differences reflect the changes of environment or the impact of historical events. Birth cohort effects refer to the risk between different birth cohorts. Different birth cohorts go through stages of life in different years and therefore different life trajectories bring them unique effects [32, 33].

2 It refers to the program of reforms called “socialism with Chinese characteristics” since 1978 and involves economic, political, cultural and other domains. In the economic domains, China has gradually established the socialist market economic system, and actively tried to attract foreign investment and develop foreign trade. In this process, the Chinese economy has achieved great achievements, but the gap between rich and poor has also been widening rapidly.
